# Use of medicines in São Paulo, Brazil, and State Health Care Coverage, 2003 and 2015

**DOI:** 10.6061/clinics/2021/e2781

**Published:** 2021-07-08

**Authors:** Camila Nascimento Monteiro, Felipe Tadeu Carvalho Santos, Karen Sarmento Costa, Marilisa Berti de Azevedo Barros, Chester Luiz Galvão Cesar, Moisés Goldbaum

**Affiliations:** I Departamento de Medicina Preventiva, Faculdade de Medicina FMUSP, Universidade de Sao Paulo, Sao Paulo, SP, BR; II Nucleo de Indicadores e Sistemas de Informacao, Hospital Israelita Albert Einstein, Sao Paulo, SP, BR; III Faculdade de Ciencias Medicas, Universidade Estadual de Campinas, Campinas, SP, BR; IV Faculdade de Saude Publica, Universidade de Sao Paulo, Sao Paulo, SP, BR

**Keywords:** Use of Medicines, Pharmaceutical Policies, State Health Care Coverage, Population Health Survey

## Abstract

**OBJECTIVES:**

To analyze the use and acquisition of medicines in São Paulo, Brazil, in 2003 and 2015, according to sociodemographic factors, socioeconomic status, and health conditions of the population.

**METHODS:**

Data were obtained from population health surveys “ISA-Capital”. Descriptive analysis, bivariate analysis, and logistic regression models were used to evaluate the use of medicines and coverage by the Brazilian Unified Health System (SUS) according to socioeconomic status and health conditions in two periods: 2003 and 2015.

**RESULTS:**

From 2003 to 2015, the surveys showed an increase in the income and education level of the study population. There was no increase in the prevalence of chronic diseases and use of medicines from 2003 to 2015. The provision of medicines by SUS was higher in 2015 than in 2003, and the coverage by SUS was higher in the population with lower education level and income in both 2003 and 2015.

**CONCLUSIONS:**

The use of medicines, mainly for chronic disease control, did not change over the years, and there was an increase in SUS coverage for medicines during 2003-2015 in all population groups, with a greater impact on the lower socioeconomic status population. The programs of the provision of medicines implanted since 2003 had influenced the greater SUS coverage for medicines and in the reduction of inequalities in access to medicines.

## INTRODUCTION

The Brazilian Unified Health System (SUS) covers the execution of therapeutic assistance operations, including the provision of some medicines (1,2). The Brazilian National Pharmaceutical Assistance Policy is organized into three components: the specialized component, which includes high-cost, specialized medicines; strategic component, which includes medicines used to treat diseases with an endemic profile and impact on the socioeconomic status; and basic component, which includes medicines to control diseases prevalent in the population and that are a priority to public health (3,4). Policies and programs have been implemented in Brazil to expand the population's access to medicines (3-6).

Among the policies and programs implemented in Brazil, we highlight the Generic Medicines Policy (1998), which has made a great contribution toward expanding the supply of medicines to the population, with a strong incentive for the production and commercialization of generic medicines in Brazil; HiperDia system (2002), which provides municipalities with an instrument that allows the monitoring of patients with hypertension and diabetes and includes the medicines used to control these diseases; and “Farmácia Popular do Brasil” program (2004) and “Aqui tem Farmácia Popular” program (2011), which emerged as an innovation for public pharmaceutical assistance policy through the adoption of co-payment for access to medicines. Moreover, in São Paulo, there are programs such as “Dose Certa” (1995) and “Remédio em Casa” (2005), which aim to improve access to medicines (4-10). São Paulo city has approximately 570 public health care services, in addition to private health care services, that are responsible for delivering medicines to the population (11,12).

Owing to the complexity of services and considering that the provision of medicines represents one of the largest expenditures in the public health system (13), the analysis of the use and acquisition of medicine by the population has become challenging. Although many studies have been conducted in the pharmaceutical assistance area (5,6,13-16), and considering the use of medicines as an eclectic collection of descriptive and analytic methods for the quantification, with many databases and technologies, there is still much to be explored in the area of the use and acquisition of medicines by the population and State Health Care Coverage of medicines.

This study aimed to analyze the use and acquisition of medicines by individuals aged ≥20 years in São Paulo in the years 2003 and 2015 according to sociodemographic factors, socioeconomic status, and health conditions of the population.

## METHODS

We used data from population-based health surveys (ISA-Capital) conducted in the city of São Paulo in 2003 and 2015, which are two cross-sectional studies. These surveys aimed to evaluate the living and health conditions of the population and use of health services.

The ISA-Capital survey used a sample that is representative of the entire non-institutionalized population from the urban area of the city of São Paulo, obtained through probabilistic sampling procedures, stratified by conglomerates in two stages: census sectors (primary sampling unit) and households (secondary stage sampling unit). The census sectors were stratified according to the socioeconomic status defined by the proportion of heads of families with different education levels (up to 5% of heads with a university-level education, from 5% to 25%, and from ≥25%).

To guarantee minimum sample sizes in population subgroups of interest, eight study domains formed by the groups in 2003 were defined: male and female individuals aged below 1 year; male and female individuals aged 1-11 years; male individuals aged 12-19, 20-59, and ≥60 years; and female individuals in the same age groups. For each of these domains, 420 interviews were planned. In 2015, domains 0-11 years did not enter the sample.

The Ethics Research Committee of the Faculty of Public Health of the University of São Paulo approved the design and conception of the ISA-Capital 2003 and ISA-Capital 2015 surveys. Interviews were conducted by trained and supervised personnel. The interviewees signed a document in which the research objectives were explained, ensuring anonymity and confidentiality of the data obtained. To ensure quality control of data collection, information on approximately 10% of the questionnaires was verified by a new interview.

The complete methodology of the ISA-Capital surveys has been described in literature (17-19).

The sociodemographic and socioeconomic characteristics of the population aged ≥20 years studied in 2003 and 2015 were as follows: age, sex, ethnicity, income, and education level. The proxy of health condition was “Acute Disease” and “Chronic Disease.” The report of a health problem in the two weeks before the interview referred to in the study was classified as “Acute Disease” and the variable “Chronic Disease” when the participant reported the presence of at least one of the following chronic diseases: arterial hypertension, diabetes mellitus, heart disease, cancer, arthritis, osteoarthritis, osteoporosis, asthma, bronchitis, rhinitis, sinusitis and any other lung disease, tendonitis, varicose veins, spine disease or spine problem, emotional or mental problem, and any other chronic disease.

A descriptive analysis of the population aged >20 years was performed, and the difference in prevalence between 2003 and 2015 was examined through a comparison of confidence intervals. When there was an overlap of intervals, the difference was not considered statistically significant.

The interviewees answered the question, “Did you use medicines in the last three days?” All medicines reported by the interviewees were listed, and then questions were asked regarding the prescription and acquisition for each medicine mentioned. Interviewers were instructed to ask for the medicine and to examine its labels when available. Logistic regression models were utilized to analyze the use of medicines three days before the interview according to socioeconomic status, sociodemographic factors, and health conditions.

Among the population that used medicine in the three days before the survey, we asked about the acquisition of these medicines. When the interviewee obtained the medicine in public health care services or through pharmaceutical assistance programs, we considered “Acquisition by SUS”; when the interviewee paid for the medicine, we considered “Obtaining “non-SUS.” Logistic regression models were used to analyze the acquisition of medicines under SUS according to socioeconomic status, sociodemographic factors, and health conditions.

## RESULTS

In total, 3,357 interviews were conducted in 2003, and 1,667 of them were conducted among individuals aged ≥20 years; 4,043 interviews were conducted in 2015, and 3,184 of them were conducted among individuals aged ≥20 years.

The characteristics of the population in 2003 and 2015 are listed in Table 1. There was an improvement in the population's income and education level from 2003 to 2015. There was an increase in the prevalence of chronic diseases during 2003-2015.

There was an upward trend in the use of medicines from 2003 to 2015, even without a statistically significant difference. In 2003, 48.82% (95% confidence interval [CI] 44.7-52.96) had used medicines in the three days before the interview. In 2015, 55.00% (95% CI 50.82-64.97) of the population had used medicines in the previous three days.

There were significant differences in the use of medicines and sociodemographic characteristics in 2003 and 2015 regarding sex, ethnicity, and “Acute Disease” (Table 2).

Among those who utilized medicines, there was an increase in obtaining these medicines from SUS compared with obtaining from “non-SUS” sources during 2003-2015.

The acquisition of medicines under SUS according to sociodemographic factors, socioeconomic status, and health conditions in 2003 and 2015 is shown in Table 3. In the model adjusted for age, sex, ethnicity, education, income, chronic disease, and “Acute disease,” the coverage of medicines under SUS was greater in the non-white population, with lower income and lower education. There was an upward trend in obtaining medicines under SUS from 2003 to 2015 across all income and education levels, even without statistically significant differences in some variables. There was an increasing tendency toward the acquisition of medicines under SUS from 2003 to 2015 across all income and education levels.

## DISCUSSION

There was an increase in the use of medicines from 2003 to 2015, although the difference was not statistically significant. There was an increase in the population's income and education level from 2003 to 2015 and an increase in the prevalence of chronic diseases during this period. The acquisition of medicines from SUS was greater in 2015 than in 2003 and was greater in the population with less education level and lower income in both 2003 and 2015.

Regarding health conditions, there was an increase in the prevalence of chronic diseases, which may be related to the expansion of primary health care, which promoted an increase in the diagnosis of all chronic diseases (15,20). There was an expansion in primary health care in São Paulo city during 2003-2015 (21).

Approximately half of the population used at least one medication in the three days before the interview, and the proportion of the population that used at least one medicine in the three days before the interview did not show a statistically significant increase: 48.8% in 2003 and 55% in 2015. According to the National Survey on Access Use and Promotion of Rational Use of Medicines Services (20), 76.2% reported having used medicines in the 30 days before the interview.

During 2003-2015, there was no expansion in the use of medicines, but only a change in the source of obtaining them: from private pharmacies to SUS. The acquisition of medicines from SUS was greater in 2015 than in 2003. The increase in SUS coverage for obtaining medicines during 2003-2015 is certainly related to the programs for the provision of medicines implemented in Brazil and the state and municipality of São Paulo, as described in the Introduction section. In addition, the distribution of medicines in the basic, strategic, and specialized components has favored access by the population, mainly the population with lower socioeconomic status.

The distribution of medicines is an integral part of health services and occurs to provide the population with greater access to this technology, intending to ensure a prophylactic, curative, palliative action or to diagnose exacerbations that affect individuals (14,15). Public drug supply programs and the growing incorporation of medicines into the health system are factors that have contributed to expanding access to these. The expansion in access to medicines in the public system has been evidenced mainly for medicines aimed at controlling chronic diseases (19,22,23) and is in line with the population's need: an increase in the prevalence of chronic diseases from 2003 to 2015 was observed in this study. According to the literature (15,19,22,23), chronic diseases are considered one of the most challenging global problems in public health and are among the main causes of death worldwide and Brazil, consequently leading to higher consumption of medicines by the population.

The population with less education level and lower income in both 2003 and 2015 was the one that sought medicines the most within the scope of SUS. Public drug supply programs promoted changes in obtaining medicines under SUS in São Paulo during 2003-2015. Public policies and programs aim to expand access to medicines from SUS and consequently to reduce inequalities in access, which, according to Jiang et al. (24), is influenced by socioeconomic factors. Brazil is a privileged setting for the debate on social inequalities, mainly because of its long tradition of commitment to equity in health (25,26).

There are many challenges in São Paulo in terms of access to health care services and the provision of medicines, including historical inequality and underfunding of the health system (26). Socioeconomic conditions can explain the great diversity in health levels; high-income inequality is associated with worse population health (25). Given its importance in the health system, it is necessary to identify inequalities in the area of medicine. According to Nunes et al. (26), inequalities can be reduced through sectoral policies, even under great social and economic gaps where the concentration of income is emblematic of the situation, a characteristic present in the case of medicines.

This study has some limitations. General access to all classes of medicines was studied, data on the acquisition of medicines by SUS according to singular classes of medicines were not analyzed. The population that needed to use medicine and did not have access was not considered in the study, given its small representation. Regarding the acquisition of medicines exclusively under SUS, there were more statistically significant associations in 2015 than in 2003, probably because of the smaller sample size in 2003.

This study provides evidence to strengthen the pharmaceutical assistance policies and programs in Brazil. Monitoring the use of medicine at two time points, i.e., 2003 and 2015, indicates that the acquisition of medicines by SUS has increased because of the policies and initiatives implemented in Brazil. Additionally, the study strengthens the role of monitoring health and health service indicators through population surveys and the necessity of continuing to conduct population-based health surveys to monitor the use of medicines and access to the health care system.

## AUTHOR CONTRIBUTIONS

Monteiro CN and Goldbaum M participated in the study conception and design, analysis and interpretation of data, and writing of the manuscript. Costa KS and Santos FTC participated in data analysis and writing of the manuscript. Barros MBA and Cesar CLG participated in the conception and design of the study and writing of the manuscript. All authors have reviewed and approved the final version of the manuscript and agree with its content and submission.

## Figures and Tables

**Table 1:** Distribution of sociodemographic characteristics, socioeconomic status, health conditions, and use of medicines in São Paulo, Brazil, in 2003 and 2015.

	2003		2015	
	% (n)	95% CI	% (n)	95% CI
Age, years				
20-39	52.04 (502)	48.79-55.27	46.15 (1,175)	43.99-48.33
40-59	31.98 (293)	29.06-35.04	35.34 (990)	33.54-37.19
≥60	15.99 (872)	14.09-18.09	18.51 (1,019)	16.56-20.63
Sex				
Male	45.09 (803)	42.01-48.20	46.26 (1,340)	44.54-47.98
Female	54.91 (864)	51.80-57.99	53.74 (1,844)	52.02-55.46
Ethnicity				
White	67.42 (1077)	63.58-71.03	50.20 (1,120)	46.51-53.87
Non-white	32.58 (542)	28.97-36.42	49.80 (1,240)	46.13-53.49
Education (years of study)				
0-7	39.84 (959)	36.83-42.93	18.93 (854)	17.17-20.82
8-11	36.23 (450)	32.7-39.92	52.46 (1,626)	49.73-55.18
≥12	23.93 (231)	20.32-37.95	28.61 (686)	25.08-32.42
Income (minimum wage)				
≤1	38.17 (689)	34.21-42.29	22.60 (728)	19.75-25.22
1>2	23.82 (443)	20.82-27.11	33.61 (935)	27.81-33.45
2<5	21.9 (355)	18.2-26.11	30.30 (886)	27.86-33.33
≥5	16.11 (180)	11.92-21.43	15.51 (412)	13.43-20.22
Chronic disease	40.11 (514)	35.66-44.72	67.19 (2,266)	64.73-69.56
Acute disease	27.91% (448)	24.05-32.13	18.95 (642)	17.39-20.62
Use of medicines	48.82 (937)	44.7-52.96	55.00 (1,936)	50.82-64.97
Acquisition of medicines by SUS	24.12 (276)	15.09-36.89	35.9 (563)	32.4-39.6

**Table 2:** Use of medicine according to sociodemographic characteristics, socioeconomic status, and health conditions in São Paulo, Brazil, in 2003 and 2015.

	Use of medicine in 2003			Use of medicine in 2015		
	n	% (95% CI)	*p*-value	n	% (95% CI)	*p*-value
Age, years			**<0.001**			**<0.001**
20-39	179	38.99 (33.98-44.25)		542	45.53 (42.10-49.01)	
40-59	145	52.01 (45.42-58.53)		630	63.41 (59.67-66.99)	
≥60	613	74.40 (70.21-78.19)		844	84.39 (81.22-87.11)	
Sex			**<0.001**			**<0.001**
Male	382	37.99 (32.69-43.59)		703	49.06 (45.91-52.22)	
Female	555	57.75 (52.64-62.70)		1313	67.63 (64.32-70.76)	
Ethnicity			**0.0060**			0.0002
White	651	53.13 (47.61-58.58)		432	53.41 (49.22-57.55)	
Non-white	284	39.79 (33.14-46.85)		526	64.97 (60.57-69.13)	
Years of study			0.0595			**<0.001**
0-7	604	51.27 (46.26-56.26)		642	70.14 (66.73-73.97)	
8-11	207	43.01 (36.78-49.46)		923	53.36 (50.50-56.20)	
≥12	116	53.55 (44.55-62.34)		439	61.93 (57.05-66.59)	
Income (minimum wage)			0.0655			**0.0061**
≤1	380	46.60 (41.31-51.96)		503	65.59 (61.65-69.23)	
1>2	240	43.16 (36.77-49.78)		569	55.13 (50.99-59.19)	
2<5	209	51.76 (44.06-59.39)		541	57.49 (53.23-61.63)	
≥5	108	58.41 (46.64-69.28)		267	61.09 (54.83-67.00)	
Chronic disease			**<0.001**			**<0.001**
No	145	27.45 (22.46-32.44)		278	29.92 (26.21-33.92)	
Yes	792	76.68 (72.55-80.52)		1738	73.20 (70.78-75.49)	
Acute disease			**<0.001**			**<0.001**
No	610	40.81 (36.07-45.73)		1463	53.53 (50.9-56.14)	
Yes	327	69.5 (63.5-74.91)		551	82.50 (78.74-85.72)	

**Table 3:** Acquisition of medicines by SUS according to sociodemographic characteristics, socioeconomic status, and health conditions. São Paulo, Brazil, 2003 and 2015.

	Acquisition of medicines by SUS 2003*				Acquisition of medicines by SUS 2015**			
	% (n)	*p*-value	Crude OR (95% CI)	Adjusted OR***	% (n)	*p*-value	OR (95% CI)	Adjusted OR***
Age, years								
20-39	27.36 (51)	0.8038	1	1	26.22 (66)	**0.0013**	1	1
40-59	26.58 (41)		1.04 (0.57-1.91)	1.03 (0.54-1.96)	37.8 (164)		**1.71 (1.70-2.50)**	0.76 (0.44-1.32)
≥60	27.39 (184)		1.17 (0.75-1.82)	1.47 (0.87-2.43)	40.87 (333)		**1.94 (1.32-2.86)**	0.65 (0.35-1.28)
Sex		0.3674				0.3413		
Female	27.64 (171)		1	1	37.72 (190)		1	1
Male	24.01 (105)		0.83 (0.54-1.25)	0.94 (0.61-1.45)	34.91 (373)		0.91 (0.87-1.41)	0.87 (0.45-1.52)
Ethnicity		**0.002**				**<0.001**		
Non-white	38.79 (125)		1	1	40.90 (118)		1	1
White	21.65 (150)		**0.43 (0.28-0.67)**	**0.58 (036-0.94)**	32.15 (127)		**0.64 (0.42-0.76)**	1.00 (0.86-1.11)
Years of study		**0.0005**				**<0.001**		
0-7	42.18 (134)		1	1	55.87 (276)		1	1
8-11	27.73 (128)		**0.20 (0.08-0.46)**	**0.31 (0.12-0.78)**	45.39 (99)		**0.63 (0.46-0.87)**	**0.57 (0.36-0.91)**
≥12	12.98 (12)		**0.52 (0.36-0.75)**	**0.55 (0.37-0.81)**	23.93 (181)		**0.25 (0.19-0.33)**	**0.26 (0.12-0.51)**
Income (minimum wage)		**0.0053**				**<0.001**		
<1	33.82 (56)		1	1	44.5 (347)		1	1
1	36.94 (106)		1.15 (0.63-2.07)	0.53 (0.26-1.19)	33.65 (133)		**0.63 (0.43-0.91)**	0.64 (0.40-1.00
2-4	26.85 (77)		0.72 (0.38-1.35)	0.69 (0.36-1.32)	22.27 (57)		**0.36 (0.25-0.49)**	0.73 (0.42-1.24)
≥5	17.17 (37)		**0.40 (0.19-0.83)**	0.95 (0.52-1.74)	17.68 (26)		**0.27 (0.16-0.46)**	0.54 (0.24-1.20)
Chronic disease		0.1753				**0.0036**		
Yes	27.85 (248)		1	1	21.91 (26)		1	1
No	21.45 (28)		0.71 (0.42-1.17)	0.86 (0.50-1.47)	37.40 (537)		2.10 (1.30-3.84)	**2.19 (1.40-3.45)**
Acute disease		0.0523				0.7181		
Yes	32.03 (111)		1	1	36.36 (387)		1	1
No	22.59 (165)		0.62 (0.38-1.00)	0.68 (0.40-1.14)	35.11 (176)		0.97 (0.76-1.30)	1.00 (0.70-1.95)

* Acquisition of medicines by SUS among the population who used medication in 2003. n=276.

** Acquisition of medicines by SUS among the population who used medication in 2015. n=563.

*** Adjusted OR: Adjusted for age, sex, ethnicity, income, education, and Chronic Disease and Acute Disease.OR, odds ratio; SUS, Brazilian Unified Health System.
